# The impact of post-exercise hydration with deep-ocean mineral water on rehydration and exercise performance

**DOI:** 10.1186/s12970-016-0129-8

**Published:** 2016-04-16

**Authors:** Douglas A. Keen, Eleni Constantopoulos, John P. Konhilas

**Affiliations:** Department of Physiology, University of Arizona, 1501 N. Campbell Ave., Tucson, AZ 85724 USA; Sarver Molecular Cardiovascular Research Program, University of Arizona, 1501 N. Campbell Ave., Tucson, AZ 85724 USA

**Keywords:** Hydration, Deep-ocean mineral water, Peak torque extension

## Abstract

**Background:**

Dehydration caused by prolonged exercise impairs thermoregulation, endurance and exercise performance. Evidence from animal and human studies validates the potential of desalinated deep-ocean mineral water to positively impact physiological and pathophysiological conditions. Here, we hypothesize that deep-ocean mineral water drawn from a depth of 915 m off the Kona, HI coast enhances recovery of hydration and exercise performance following a dehydrating exercise protocol compared to mountain spring water and a carbohydrate-based sports drink.

**Findings:**

Subjects (*n* = 8) were exposed to an exercise-dehydration protocol (stationary biking) under warm conditions (30 °C) to achieve a body mass loss of 3 % (93.4 **±** 21.7 total exercise time). During the post-exercise recovery period, subjects received deep-ocean mineral water (Kona), mountain spring water (Spring) or a carbohydrate-based sports drink (Sports) at a volume (in L) equivalent to body mass loss (in Kg). Salivary samples were collected at regular intervals during exercise and post-exercise rehydration. Additionally, each participant performed peak torque knee extension as a measure of lower body muscle performance. Subjects who received Kona during the rehydrating period showed a significantly more rapid return to pre-exercise (baseline) hydration state, measured as the rate of decline in peak to baseline salivary osmolality, compared to Sports and Spring groups. In addition, subjects demonstrated significantly improved recovery of lower body muscle performance following rehydration with Kona versus Sports or Spring groups.

**Conclusions:**

Deep-ocean mineral water shows promise as an optimal rehydrating source over spring water and/or sports drink.

## Findings

### Background

High-intensity exercise in athletes and regularly active individuals typically results in dehydration that, if sufficiently severe, impacts physical performance and mental capacity [1]. Moreover, a hot environment amplifies dehydration and performance deficits. Interestingly, animals supplemented with desalinated deep-ocean mineral water show benefit on physiological and pathophysiological conditions [2–5]. Similarly, a recent study shows that deep-ocean water (662 m) taken from the coast of Hualien, Taiwan, increases recovery following dehydrating exercise, evidenced by accelerated recovery of aerobic capacity, increased lower-body muscle power performance, and significantly reduced levels of exercise-induced muscle damage markers over subjects drinking purified tap-water [5]. These data suggest that deep-ocean mineral water may provide optimal rehydration for performance following high-intensity exercise in well-conditioned individuals. Therefore, development of an efficient and optimal rehydration strategy could prove beneficial during high-intensity activities. To further explore this supposition, we tested the hypothesis that rehydration with deep-ocean water (915 m) drawn from Kona, HI (Kona Deep™) at the Hawaii Ocean Science and Technology Park (National Energy Laboratory of Hawaii Authority) will improve post-exercise performance over mountain spring water or a carbohydrate-based sports drink.

## Methods

Well-conditioned student-athletes free from alcohol, medication or major health-related issues determined by a health questionnaire were included in the study (age: 23 ± 1.2 years, height: 175.6 ± 6.0 cm, weight: 76.7 ± 8.0 Kg). Subjects were randomized to 1 of 3 experimental groups, Kona (*n* = 6), Sports (*n* = 8), or Spring (*n* = 6). With a conservative estimate of salivary osmolality at the beginning (100 mmol/Kg) and end (150 mmol/Kg) of the experimental protocol with a standard deviation of 30, sample size was determined using a power of 0.8 at α = 0.05 (6 or greater). All subjects provided consent under protocols adhered to guidelines approved by the Institutional Review Board at the University of Arizona and in accordance with the Declaration of Helsinki. Subjects were asked to follow a normal diet while avoiding foods high in sodium 24 hours prior to study initiation. Euhydrated subjects fitted with a Polar™ heart rate monitor were exposed to an exercise-challenge (stationary biking) under warm conditions (30 °C) because sweat rate is highest at warm ambient temperatures (31 °C) [6]. Subjects were required to maintain 150–200 watts on the stationary bicycle. Target dehydration was a body mass loss of 3 % where a similar study using deep ocean mineral water demonstrated a significant exercise deficit [1, 5, 7]. Body mass measurements were taken prior to exercise and then at 15-minute intervals throughout the exercise trial and during rehydration. Also at each interval, stimulated saliva was collected and salivary osmolality was measured by a vapor pressure osmometer (Wescor model 5600 [8]) as a measure of hydration status [9, 10]. Subjects completed the exercise protocol in 93.4 ± 21.7 minutes total exercise time (range: 60–135 minutes) with no differences among experimental groups.

Upon reaching the appropriate body mass loss, subjects were required to consume a volume matching body mass loss such that total volume of intake was equal to body mass loss assuming 1 liter = 1 kg. Due to the volume of fluid required for the rehydration protocol, subjects were asked to rehydrate in two phases to prevent hypervolemia. During the first rehydrating phase, subjects consumed one-half of the total volume immediately following completion of the exercise protocol. Salivary samples were collected starting at 10 minutes following initial rehydrating phase and then every 5 minutes. At 30 minutes following the initial rehydrating phase, subjects consumed the remainder of the rehydrating fluid. Salivary samples were collected starting at 10 minutes and then every 5 minutes up to 45 minutes following the second rehydrating phase.

Study subjects performed a series of 3 maximal contractions of the left knee extensors using a Biodex™ dynamometer to measure of peak torque extension prior to exercise, immediately after exercise, and after the final rehydrating phase.

## Results and Discussion

**Fig. 1:**
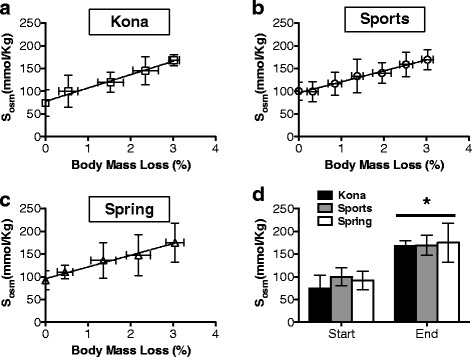
Rate of dehydration and rehydration in subjects challenged with a dehydrating exercise protocol. The change in salivary osmolality (S_osm_ mmol/Kg) relative to body mass loss was not different among study subjects whether placed into the Kona (**a**), Sports (**b**) or Spring (**c**) experimental group. Data presented as binned samples ± standard deviation (S.D.). **d** Bar graph representation for salivary osmolality (S_osm_ mmol/Kg) in each group prior to (**Start**) and immediately following (**End**) dehydration exercise protocol. Salivary osmolality was significantly higher at the end of the exercise protocol compared to each respective group at the start of the exercise protocol (*p < 0.05). Data presented as ± S.D

In all experimental groups, stimulated salivary osmolality (mmol/Kg) increased with body mass loss during the exercise protocol and there were no significant differences in the rate of body mass loss or peak stimulated salivary osmolality at the end of the dehydration protocol (Fig. 1a). Previous work shows that salivary osmolality is the most effective index during activity [10]. The exercise protocol was stopped when subjects reached a 3 % body mass loss and the rehydrating phase was initiated. The rate of recovery to baseline salivary osmolality for each subject was determined. Subjects rehydrating with deep-ocean water (Kona) demonstrated a more rapid return (2-fold) to baseline salivary osmolality (Fig. 1b) when compared to all other groups. Previous work demonstrated that return to pre-exercise plasma sodium was better achieved with a sodium-containing (a sports drink similar to that used in this study) over a sodium-free (water) beverage [11]. Although the deep-ocean mineral water (Kona) contains a greater amount of sodium, potassium, calcium and magnesium compared to mountain spring water (Spring), the carbohydrate-based beverage (Sports) has almost 10-fold greater amounts, and consequently, higher osmolality, of these components. Our data suggests that Kona accelerates the return of salivary content over a carbohydrate-based electrolyte solution (Sports) or mountain spring water (Spring). Interestingly, the composition of the intake fluid impacts intestinal water flux more so than osmolality [12].

**Fig. 2:**
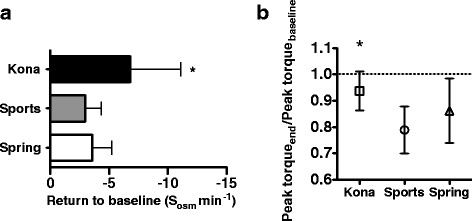
Impact of rehydration fluid on salivary osmolality and lower body muscle performance following rehydration. **a** The rate of return to baseline was determined by best-fit line for each trial from peak salivary osmolality to post-hydration salivary osmolality that was equivalent baseline salivary osmolality. The slopes for each individual were then averaged and compared by ANOVA followed by Tukey’s multiple comparison test (*p < 0.05). **b** Peak torque extension was determined prior to and immediately following exercise protocol, and then 75 minutes post exercise and following complete rehydration of the study fluid. Subjects rehydrating with Kona showed a more significant return to baseline performance over Sports and Spring. Data presented as mean ± S.D. and compared by ANOVA followed by Tukey’s multiple comparison test (*p < 0.05)

Because body mass loss due to fluid depletion suppresses exercise performance [1], we implemented the peak torque extension as a measure of lower body muscle performance. We chose the peak torque extension maneuver over more extensive, fatiguing-based tests such as maximal oxygen consumption because we wished to more closely align performance deficit with the dehydrating protocol and minimize the performance deficit from the performance measure itself. Consequently, a brief measure of peak power production that was repeated at least three times during the full protocol was chosen as an optimal measure of performance. Peak torque during rapid leg extension was determined prior to and immediately following the exercise protocol, and then 75 minutes post exercise following complete rehydration of the study fluid. Although subjects in each study group failed to fully recover baseline peak torque extension following rehydration, the Kona group achieved the highest return to baseline that was statistically significant compared to Sports or Spring groups (Fig. 2). The beneficial effects of deep-ocean mineral water may be attributed to its unique mineral composition. Depletion of these essential minerals, including sodium, calcium, potassium and magnesium may lead to reduced neuromuscular function and physical performance [1, 13].

### Conclusions and future directions

While it is clear that dehydration due to physical exertion such as exercise impairs thermoregulation, endurance and exercise performance, proper rehydrating strategies to counteract these effects have yet to be clearly elucidated. Studies regarding the use of deep-ocean mineral water as a potential alternative to rehydration with water or high-carbohydrate high-electrolyte sports drinks are limited with only two studies (one animal [14] and one human [5]) reported in the literature. Our findings from this study support the utility of Kona as an essential part of a rehydrating strategy to optimize exercise performance over mountain spring water or carbohydrate-based sports drinks. We also suggest that the recovery of exercise performance post-exercise may be due to the finding that Kona accelerates a return to baseline salivary osmolality from peak values at the end of exercise. Nevertheless, questions remain as to the mechanisms by which deep-ocean mineral water promotes a more rapid recovery in exercise performance and rehydration. For example, the three rehydrating fluids contain different amounts of sodium, potassium, calcium and magnesium, yet how these differences underlie the unique hydrating properties of each beverage post-exercise remains unknown. Furthermore, it is not known whether Kona enhances exercise performance under non-dehydrating conditions. Future studies will be directed at these and other questions.
